# Teaching health professionals how to tailor gender-affirming medicine protocols: A design thinking project

**DOI:** 10.1007/s40037-020-00581-5

**Published:** 2020-04-16

**Authors:** Kinnon R. MacKinnon, Lori E. Ross, David Rojas Gualdron, Stella L. Ng

**Affiliations:** 1 Dalla Lana School of Public Health, University of Toronto, Toronto, Ontario Canada; 2 The Wilson Centre, Faculty of Medicine at University Health Network, University of Toronto, Toronto, Ontario Canada; 3 Centre for Ambulatory Care Education, Women’s College Hospital, Toronto, Ontario Canada

**Keywords:** Protocols, Gender-affirming medicine, Continuing education, Design thinking

## Abstract

**Background:**

Content knowledge surrounding transgender (trans) medicine is currently lacking in the formal medical education curricula. Evidence indicates that the main protocols used to assess and refer trans patients for gender-affirming medicine are misunderstood by health professionals, and require flexible adaptation to achieve health equity and patient-centred care.

**Approach:**

A free online educational tool for gender-affirming medicine, *The Path to Patient-Centred Care*, was developed to teach learners how to adapt assessment protocols. Resource creation was supported by a knowledge translation grant that endorsed design thinking, a human-centred and solutions-focused framework recommended for use in curriculum development.

**Evaluation:**

*The Path to Patient-Centred Care* provides learners with information related to key principles of patient-centred care in gender-affirming medicine, including a guide on how to adapt the main assessment protocols to achieve equitable care. The curriculum also includes narratives from trans patients and health professionals that focus on health equity, and a clinical vignette about a complex case, designed to foster critical thinking on medical ethics. Project future directions involve an implementation and evaluation pilot study with a diverse group of continuing professional development medical learners using a mixed-methods program evaluation design.

**Reflection:**

The use of design thinking to develop this resource exemplifies a novel approach to curriculum development. By using pedagogical strategies that foster critical reflection, this innovative online education tool strives to teach self-directed learners how to provide care that emphasizes trans people’s self-determination and autonomy in medical decision-making.

## Background and need for innovation

Protocols pervade medical practice yet they stir much debate in medical education and health services research. Health professionals caring for transgender (trans) patients in the context of gender-affirming medicine are particularly reliant on medical protocols, which guide assessments related to hormones and surgeries. Gender-affirming medicine includes hormones (e.g., oestrogen, progesterone, antiandrogens, and testosterone), chest/breast surgery (e.g. breast augmentation and chest reconstruction surgeries), and genital surgery (e.g., orchiectomy, vaginoplasty, hysterectomy, metoidioplasty, and phalloplasty) [1]. Trans people face numerous barriers to gender-affirming medicine. Medical education gaps are cited as a major barrier to healthcare for trans people [2–4]. A review of trans medical education programs found that when offered at all, these are limited to one-time awareness-based interventions and rarely include advanced clinical practice skills such as gender-affirming medicine [4]. Finding clinicians who can compassionately provide gender-affirming medicine proves especially challenging, which may exacerbate mental health challenges in this population [1, 5]. While not all trans people seek gender-affirming medicine, those who do rely on health professionals with the knowledge and skills to use gender-affirming medicine assessment protocols.

The World Professional Association for Transgender Health (WPATH) standards of care [6] serves as a form of curriculum—teaching a standard of care in gender-affirming medicine [7, 8]. The WPATH standards of care provide the main protocols for assessing patient readiness for gender-affirming medicine [6]. It is important to note that social science scholarship indicates several problems with these standards. First, they are misunderstood by health professionals due to the absence of formal education and training [8]. Second, given that health professionals apply assessment criteria to determine when, or if, trans patients can access gender-affirming medicine, a relationship exists between the WPATH standards of care protocols and medical paternalism [7, 8]. These protocols contribute to poor patient-provider alliances because trans patients feel compelled to strategically present narratives according to assessment criteria in order to mitigate the risk of being denied gender-affirming medicine [8, 9]. Furthermore, it is argued that gender-affirming medicine protocols do not meet *Oxford Centre for Evidence-Based Medicine* criteria, despite claiming to be ‘evidence-based’ [10]. In response to these concerns, an international movement calls for better practice to advance autonomy and self-determination for trans people [11].

Previous research shows that when encountering problems with gender-affirming medicine protocols, clinicians have informally learned how to adapt, tailor, and work around protocols, to achieve justice and equity for trans people [7, 10]. Furthermore, content knowledge related to trans people and gender-affirming medicine is currently lacking in formal medical school, residency, and continuing professional development curricula [2–4]. Numerous studies have shown positive associations between exposure to baseline knowledge about trans people and improvements in learners’ attitudes, yet pedagogical interventions focused on integrating advanced clinical skills are absent [4]. In this report we discuss the creation of a free online education tool designed to fill this education and clinical skills gap titled: *The Path to Patient-Centred Care *(PPCC) [12]. Our resource aims to teach clinicians how to offer patient-centred care to trans adults seeking gender-affirming medicine in Canada; however, its implications are relevant in all geographic locations where health professionals use the WPATH standards of care.

**Fig. 1:**
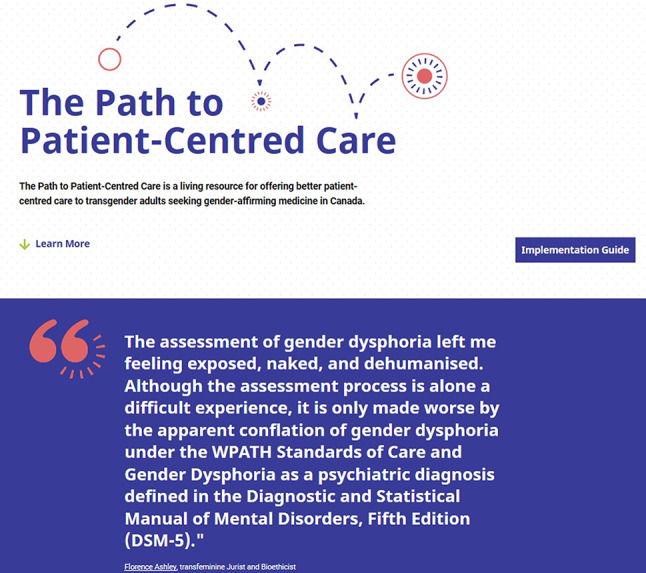
The PPCC landing page presents the learner with a quoted trans patient or health professional narrative. Different narratives (*n* *=* 9) appear as the learner transitions through the curriculum. The Implementation Guide curriculum is easily accessible for the learner

Our project studies gender-affirming medicine assessment learning and teaching in three distinct phases. Phase one study findings have been published elsewhere [7]. In the first phase of our qualitative study we found that health professionals identified problems with strict gender-affirming medicine protocols. Study participants expressed discomfort with diagnosing patients with gender dysphoria because doing so rendered patients’ identities as mentally disordered [7]. This finding is also consistent with one trans person’s experience presented in the PPCC resource shown in Fig. 1. In response, protocols were applied flexibly. In this report we outline the process of stage two: by applying design thinking, a solution-based approach to solving problems, we developed an online gender-affirming medicine education tool [13].

## Goal of innovation

The PPCC resource aims to teach learners about patient autonomy and shared decision-making with trans patients in the context of gender-affirming medicine, and to mitigate ethical tensions associated with strict application of psychosocial readiness assessments. A secondary goal is to teach learners critical reflection skills. Critical reflection refers to the examining of individual and societal assumptions, inequitable power relations, and how these interact to influence practice [14]. The PPCC educational resource provides learners with free and independently available information on how to tailor and adapt assessment protocols, for the explicit purpose of providing equitable gender-affirming medicine grounded in patient-centred care values such as patient collaboration. For the purpose of attaining this twofold educational goal, two well-known medical concepts were applied: informed consent and patient-centred care. In gender-affirming medicine, the ‘informed consent model’ emphasizes trans patient autonomy, advocating for trans people to access treatments through informed decision-making, and without the need for strict readiness assessments and a gender dysphoria diagnosis [6, 16]. ‘Patient-centred care’ redefines the traditional relationships between patients and health professionals, stressing collaboration, autonomy, and patient self-determination [15]. Patient-centred care in gender-affirming medicine is currently lacking due to the constraints of strict adherence to the WPATH standards of care assessment protocols which limit trans patient collaboration in medical decision-making [7, 10]. According to the WPATH standards of care, trans patients must be diagnosed with gender dysphoria and meet other psychosocial readiness criteria prior to treatment, which undermines patient self-determination and autonomy [16].

## Steps taken for development and implementation of innovation

Resource creation was supported by a knowledge translation and exchange initiative focused on addressing lesbian, gay, bisexual, transgender, queer (LGBTQ) health and wellness, funded by the Institute of Gender and Health, Canadian Institutes of Health Research. A key condition of this knowledge translation grant included participation in a two-day ‘Design Jam’ held in Vancouver, Canada. Author, KMK, participated in the Design Jam alongside other grantees, designers, and end knowledge users (e.g. health professionals, educators, healthcare institution stakeholders, LGBTQ people). The purpose of the Design Jam was to train grantees to apply design thinking to solve problems affecting the slow translation of LGBTQ health research into clinical practice.

Design thinking has been endorsed for use in health professions education and curriculum development, but has been rarely applied [13]. Design thinking is widely used in business, service design, and social policy, and increasingly draws attention from health professions education due to its utility for implementing and adjusting curricula [13]. Design thinking emphasizes understanding target users through its ‘human-centred’ approach, a crucial step in designing an educational tool for use in gender-affirming medicine [13]. Design thinking endorses problem solving and solutions development in five stages: 1) empathize with users; 2) define user’s problems and needs; 3) ideate through creating novel solutions; 4) prototype solutions; and 5) test possible solutions. We worked through these stages which resulted in the creation of the PPCC—translating the findings of phase one of our study and filling a medical education curriculum gap.

We additionally sought guidance from two family physicians with expertise in gender-affirming medicine, and one psychologist who teaches learners how to adapt and tailor assessment protocols. Health professionals’ insights were solicited at various stages of development, and provided important end knowledge user feedback. In the following section we describe the key content knowledge developed through the design thinking process. This curriculum was designed to teach learners how to tailor gender-affirming medicine protocols to respond to the needs of trans patients ethically and equitably.

## Outcomes of innovation

Drawing from Google Analytics data, over 1000 new users have accessed the online tool since launching in July 2019. Users have logged in from North America, Europe, and Australia. The PPCC is defined by accessibility, flexibility, and portability. Content is freely available to learners at all stages of education. The resource and its curricula could be delivered in multiple education settings such as in classrooms, workshops, or in a webinar. The resource also includes quoted narratives (*n* = 9) from trans patients and health professionals that focus on topics related to self-determination and autonomy in gender-affirming medicine (see Fig. 1). A clinical vignette about a complex case designed to foster discussion and critical reflection surrounding medical ethics and health equity is also presented. References to peer-reviewed evidence are provided throughout the PPCC. The resource is separated into sections that teach the following curricula:

### What is informed consent/patient-centred care?

This section provides learners with a brief history and rationale for the development of the informed consent model that emerged in the United States in response to problems with the traditional WPATH standards of care. Key principles associated with patient-centred care and informed consent in gender-affirming medicine, such as autonomy, self-determination, and protocol workarounds, are also explained.

### Why informed consent?

Although the WPATH standards of care presents itself as a ‘flexible’ guideline that can be tailored to patients’ individual needs, learners rarely have the skills to make protocol adaptations [6]. This section provides learners with rationale surrounding the need to tailor assessment protocols to achieve ethically sound care for trans patients.

### Implementation guide (step-by-step guides to hormones and surgeries)

Crucially, this section presents learners with the traditional assessment criteria, followed by concrete guidance on how to adapt protocols according to the values of informed consent and patient-centred care.

### Inform change

Acknowledging the value of connecting learners with other health professionals and healthcare organizations engaged in the area of gender-affirming medicine, this section serves as a placeholder for further engagement. Relevant news articles related to advancing gender-affirming medicine published in the popular press are also presented.

## Critical reflection on innovation

Our use of the design thinking approach to develop the PPCC tool exemplifies a novel strategy for curriculum development. Yet its procedural limitations must be noted. Given the knowledge translation and exchange Design Jam event occurred in February 2018 and the resource was launched in July 2019, we note that design thinking, and conducting consultation with end knowledge users, may add significant time and resource constraints to be mindful of when planning for future design thinking projects in health professions education. We also note that the development of this online resource draws largely from a transformative education paradigm, and to a lesser extent, humanism [17]. In using pedagogical strategies that foster critical reflection, the PPCC online education tool strives to teach self-directed learners how to provide gender-affirming medicine that emphasizes trans people’s self-determination and autonomy in medical decision-making. Phase three of this project involves a mixed-methods program evaluation with continuing professional development learners. While we note the pitfalls of empirically assessing learners’ critical reflections, [14] we are particularly interested in evaluating how learners gain gender-affirming medicine content area knowledge, critical reflection skills, and comfort with tailoring protocols to achieve collaborative care with trans patients. We predict that learners exposed to the PPCC online resource will become comfortable tailoring these protocols, learning critical reflection skills in turn.
