# Correction to: Teaching health professionals how to tailor gender-affirming medicine protocols: A design thinking project

**DOI:** 10.1007/s40037-020-00583-3

**Published:** 2020-05-14

**Authors:** Kinnon R. MacKinnon, Lori E. Ross, David Rojas Gualdron, Stella L. Ng

**Affiliations:** 1 Dalla Lana School of Public Health, University of Toronto, Toronto, Ontario Canada; 2 The Wilson Centre, Faculty of Medicine at University Health Network, University of Toronto, Toronto, Ontario Canada; 3 Centre for Ambulatory Care Education, Women’s College Hospital, Toronto, Ontario Canada

**Erratum to:**

**Perspect Med Educ 2020**

10.1007/s40037-020-00581-5

The original version of this article unfortunately contained a mistake. The name of David Rojas Gualdron was presented incorrectly in the author list and in the conflict of interest. The corrected author list is given above. The original article has been corrected.

